# Relación del intervalo QT corregido con la escala GRACE en pacientes con infarto de miocardio sin elevación del segmento ST

**DOI:** 10.47487/apcyccv.v3i3.223

**Published:** 2022-09-30

**Authors:** Guillermo Cruz-Aragón, Manlio F. Márquez, Angel Cueva-Parra, Héctor González-Pacheco, Pedro Iturralde, Santiago Nava

**Affiliations:** 1 Departamento de Electrofisiología, Instituto Nacional de Cardiología Ignacio Chávez. Ciudad de México, México. Departamento de Electrofisiología, Instituto Nacional de Cardiología Ignacio Chávez Ciudad de México México; 2 Unidad Coronaria, Instituto Nacional de Cardiología Ignacio Chávez. Ciudad de México, México. Unidad Coronaria, Instituto Nacional de Cardiología Ignacio Chávez Ciudad de México México

**Keywords:** Infarto del Miocardio, Electrocardiografía, Riesgo, Myocardial infarction, Electrocardiography, Risk

## Abstract

**Antecedentes.:**

El modelo de predicción del registro global de eventos coronarios agudos (GRACE por sus siglas en inglés) es usado para estratificar el riesgo en pacientes con infarto de miocardio sin elevación del segmento ST (IAMSEST). El intervalo QT corregido (QTc) no se considera en este modelo.

**Objetivo.:**

Evaluar la relación entre el intervalo QTc con la escala GRACE en pacientes con IAMSEST.

**Materiales y métodos.:**

Se realizó un estudio observacional retrospectivo entre 2016 y 2019. Se incluyeron pacientes con diagnóstico de IAMSEST, los intervalos QTc se calcularon con la fórmula de Bazett y se clasificaron en dos grupos: intervalo QTc normal (<440 ms) y prolongado (≥440 ms). Según el puntaje GRACE fueron clasificados en tres rangos: riesgo bajo (≤109 puntos), intermedio (110-139 puntos) y alto (≥140 puntos), se determinó si existía relación entre el intervalo QTc y la puntuación GRACE.

**Resultados.:**

Durante el período mencionado ingresaron en nuestro centro 940 pacientes con diagnóstico de IAMSEST, 634 cumplieron con los criterios de inclusión; hubo 390 pacientes con intervalo QTc normal y 244 con intervalo QTc prolongado. Los pacientes con QTc prolongado eran mayores (65,5 vs. 61, p=0,001) con menor proporción de hombres (71,7% vs. 82,8%, p=0,001). Se encontró asociación entre la escala GRACE y el intervalo QTC, los sujetos con un QTc normal tenían una mayor proporción de riesgo bajo e intermedio que aquellos con un QTc prolongado (p=0,001).

**Conclusiones.:**

En pacientes con IAMSEST un intervalo QTc normal (<440 ms) se relaciona con una escala de riesgo GRACE de riesgo bajo o intermedio.

## Abstract (en)

**Background.:**

The Global Registry of Acute Coronary Events (GRACE) prediction model stratifies patients with non-ST-segment elevation myocardial infarction (NSTEMI). Corrected QT interval (QTc) is not considered in this model.

**Objective.:**

To evaluate the relationship between the QTc interval and the GRACE score in patients with NSTEMI.

**Materials and methods.:**

An observational, retrospective study was carried between 2016 and 2019. We included patients with diagnosis of NSTEMI, QTc intervals were calculated with Bazett's formula, and they were classified into 2 groups: a normal QTc interval (<440 ms) and prolonged (≥440 ms). According to the GRACE score they were classified in three ranges: low risk (≤109 points), intermedium (110 - 139 points) and high (≥140 points), we determined if there were a correlation between QTc interval and the GRACE score.

**Results.:**

A total of 940 patients with a diagnosis of NSTEMI were admitted in our institution, 634 met the inclusion criteria, there were 390 patients with normal QTc interval and 244 with a prolonged QTc interval. Patients with prolonged QTc were older (65.5 vs 61, p=0.001) with a lower proportion of males (71.7% vs 82.8%, p=0.001). An association was found between the GRACE score and the QTC interval, subjects with a normal QTc had a greater proportion of low and intermediate risk than those with a prolonged QTc (p=0.001). **Conclusions.** In NSTEMI patients, a normal QTc interval (<440 ms) is associated with a GRACE risk score of low or intermediate risk.

## INTRODUCCIÓN

El Infarto agudo de miocardio sin elevación del segmento ST (IAMSEST) es una entidad de presentación heterogénea, en la que existen pacientes con características de alto riesgo que podrían beneficiarse de una estratificación invasiva temprana ^1^.

Según las guías, se recomienda el uso de escalas de riesgo como la escala GRACE (*Global Registry of Acute Coronary Events*) en pacientes con IAMSEST. Este modelo calcula el riesgo de mortalidad intrahospitalaria y el riesgo de mortalidad a los 6 meses, empleando variables como la edad, frecuencia cardiaca, tensión arterial, creatinina, alteraciones en el segmento ST, valores de enzimas cardiacas y clase de Killip y Kimball ^2^^,^^3^.

Por otro lado, múltiples estudios han establecido la utilidad del intervalo QTc como predictor de riesgo de enfermedad coronaria, muerte súbita cardiaca y mortalidad cardiovascular ^4^^-^^11^. Pese a lo anterior, el intervalo QTc no es una variable que esté incluida en la escala GRACE. Por lo que deseamos averiguar si existe relación entre la medición del mencionado intervalo y la escala GRACE en pacientes con IAMSEST.

## MATERIALES Y MÉTODOS

### Tipo de estudio y pacientes

Estudio retrospectivo, observacional, unicéntrico, entre enero de 2016 y agosto de 2019, en donde se incluyeron pacientes mayores de 18 años con diagnóstico clínico de IAMSEST, admitidos en la unidad de cuidados coronarios de nuestro centro, a quienes se les tomó un electrocardiograma (ECG) de doce derivadas, convencional (calibración 25 mm/s, 10 mm/mV).

Se excluyeron pacientes con algún trastorno del ritmo y/o de la conducción que pudiera alterar el intervalo QT: bloqueo auriculoventricular (BAV) de segundo y tercer grado; bloqueo de rama derecha (BCRD); bloqueo completo de rama izquierda (BCRI); portadores de dispositivos de estimulación cardiaca; pacientes que presentaron taquicardia ventricular (TV) al ingreso, ya que está altera el intervalo QT; fibrilación auricular (FA) y *flutter* auricular (FLA). También se excluyeron otras causas que podrían alterar el intervalo QT como: hipercalemia (5,5 mEq/L) o hipocalemia (<3,0 mEq/L), uso de fármacos que prolonguen el QT y pacientes con síndrome de QT largo congénito.

### Medición del intervalo QT

La medición del intervalo QT se realizó en el ECG de admisión del paciente, inicialmente se realizó de manera automática por el electrocardiógrafo (Welch Allyn modelo CP 200) y posteriormente se corroboró con una medición manual del intervalo QT en las derivadas DII y V5. Para la corrección del intervalo QT se empleó la fórmula de Bazett ^12^. Los pacientes fueron clasificados en dos grupos: aquellos con un intervalo QTc normal (<440 ms) y aquellos con un intervalo QTc prolongado (≥440 ms). Establecimos ese punto de corte basados en los resultados de un metaanálisis de estudios epidemiológicos realizados en la población general ^7^ y en un estudio publicado anteriormente ^11^.

### Cálculo de la escala GRACE

La determinación de la escala GRACE fue realizada de manera convencional ^3^. Para su cálculo se consideraron los valores de frecuencia cardiaca y de tensión arterial obtenidos al momento de la admisión; asimismo, se emplearon los primeros valores obtenidos de creatinina y enzimas cardiacas. Tras el cálculo de la escala GRACE los pacientes se clasificaron en tres grupos: bajo riesgo (≤ 109 puntos), riesgo intermedio (110 - 139 puntos) y alto riesgo (≥ 140 puntos).

### Análisis estadístico

Las variables cuantitativas fueron clasificadas como paramétricas o no paramétricas, dependiendo de su normalidad, la cual fue calculada por la prueba de ajuste de Kolmogorov-Smirnov. Las variables cuantitativas paramétricas fueron reportadas con media y desviación estándar, mientras que las no paramétricas fueron reportadas con mediana y rangos intercuartílicos. Asimismo, para el análisis bivariado se utilizó la prueba T de Student o U de Mann-Whitney, de acuerdo con el tipo de distribución.

Las variables cualitativas se describieron por medio de porcentajes y frecuencias, para su análisis bivariado se utilizó prueba de chi cuadrado (X²) o F de Fisher, dependiendo del número de eventos recopilados. En todos los análisis se consideró como significativo un valor de p < 0,05. Para el análisis estadístico se utilizó el programa IBM SPSS STATISTICS.

## RESULTADOS

### Pacientes

**Figura 1:**
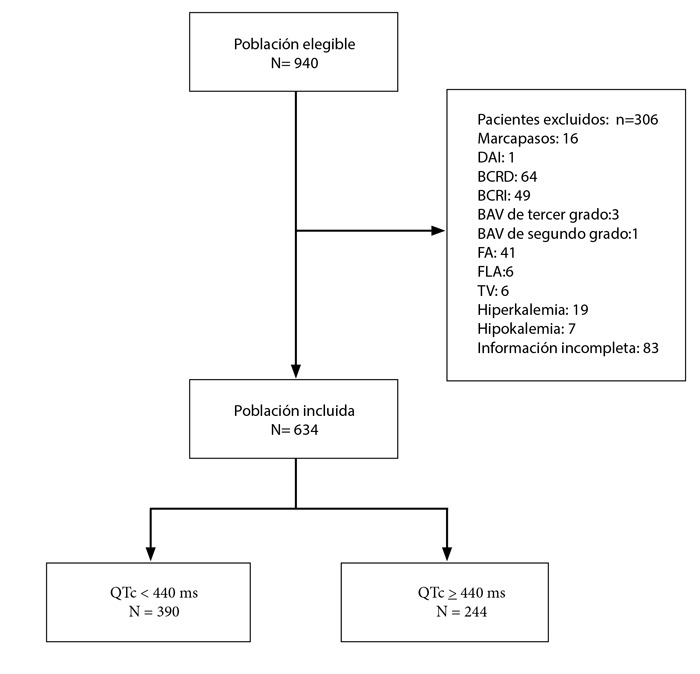
Población elegible, criterios de selección y grupos de estudio.

Durante el periodo anteriormente mencionado, 940 pacientes con diagnóstico de IAMSEST fueron admitidos a la unidad de cuidados coronarios. Se excluyeron 306 pacientes: 64 con BCRD; 49 con BCRI; 13 con BAV de tercer grado; 1 con BAV de segundo grado; 41 con FA; 6 con FLA; 6 con TV; 16 portadores de marcapasos; 1 portador de DAI; 19 con hipercalemia; 7 con hipocalemia y 83 pacientes por tener información incompleta. Finalmente, solo 634 pacientes cumplieron los criterios de selección, de los cuales 390 presentaron un intervalo QTc normal (<440 ms) y 244 un intervalo QTc prolongado (≥ 440 ms) (Figura 1).

### Características basales

La edad muestra una mediana de 63,2 años, RIQ (54-72) y el 78,5% fueron varones. Las comorbilidades más frecuentes fueron: hipertensión (65,5%), diabetes *mellitus* (42,4%) y dislipidemia (36,9%). Además, un 39,3% tuvo infarto previo y la fracción de eyección de ventrículo izquierdo (FEVI) promedio fue de 51,46%.

Al comparar ambos grupos, encontramos diferencias significativas en relación con la edad y el sexo; aquellos que presentaban un intervalo QTc ≥ 440 ms eran mayores (65,5 años vs. 61,0 años, p=0,001) y con menor proporción de pacientes de sexo masculino (71,7% vs. 82,8%, p=0,001). Con respecto a la FEVI, esta fue menor en los pacientes con intervalo QTc ≥ 440 ms (49% vs. 53%, p=0,001). No se encontraron diferencias significativas para otras variables (Tabla 1).

**Tabla 1:** Características basales de la población

Variable	QTc < 440 ms (n=390)	QTc ≥ 440 ms (n=244)	Valor p
Edad (años)	61 (54-68)	65,5 (58-72)	0,001
Varones	322 (82,8%)	175 (71,7%)	0,001
Hipertensión arterial	246 (63,1%)	169 (69,3%)	0,111
Diabetes *mellitus*	158 (40,5%)	111 (45,5%)	0,217
Dislipidemia	147 (37,7%)	87 (35,7%)	0,605
Infarto previo	149 (38,2%)	100 (41 %)	0,486
FEVI	53% (45-58)	49% (40-55)	0,001

La edad y la FEVI (fracción de eyección de ventrículo izquierdo), se encuentran reportadas como mediana y con rangos intercuartílicos.

### Parámetros de laboratorio

Para evaluar las diferencias entre los dos grupos en estudio se consideraron los primeros resultados de laboratorio, los cuales fueron tomados a la admisión del paciente. Se encontraron diferencias estadísticas significativas en determinados parámetros de laboratorio. Los pacientes con intervalo QTc ≥ 440 ms tenían niveles más elevados de troponina I (6,2 ng/mL vs. 4,3 ng/mL, p=0,006) y de NT - pro-BNP (1781 pg/mL vs. 771 pg/mL, p=0,001). No se encontraron diferencias estadísticamente significativas con respecto a los valores de lactato (1,3 mmol/L vs. 1,2 mmol/L, p=0,073) (Tabla 2).

**Tabla 2:** Parámetros laboratoriales

Variable	QTc < 440 ms (n=390)	QTc ≥ 440 ms (n=244)	Valor p
Troponina I (ng/mL)	4,3 (1,01-12,65)	6,2 (1,4-22,15)	0,006
NT-pro BNP (pg/mL)	771 (222-2167)	1781 (684-5820)	0,001
Lactato (mmol/L)	1,2 (1,0-1,6)	1,3 (1,0-1,8)	0,073

Los valores se encuentran reportadas como mediana y con rangos intercuartílicos.

### Relación con la escala GRACE y mortalidad

**Figura 2:**
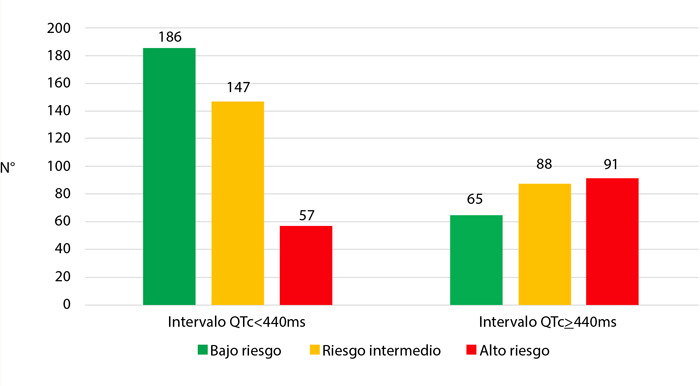
Relación entre el intervalo QT y la escala GRACE.

En el análisis comparativo de la escala de riesgo GRACE se observaron diferencias estadísticamente significativas en los tres rangos prestablecidos (≤109, 110 - 139, ≥140). La proporción de pacientes de bajo riesgo fue mayor en el grupo con intervalo QTc normal (47,7% vs. 26,6%, p=0,001). Por otro lado, la proporción de pacientes de alto riesgo fue mayor en el grupo con intervalo QTc prolongado (14,6% vs. 26,6%, p=0,001) (Tabla 3, Figura 2).

**Tabla 3:** Relación entre el QTc y la escala GRACE

Escala GRACE	QTc < 440 ms	QTc ≥ 440 ms	Total	Valor P
Bajo riesgo <109 puntos	186 (74,1%)	65 (25,9%)	251	0,001
Riesgo moderado 110 - 139 puntos	147 (62,6%)	88 (37,4%)	235	
Alto riesgo ≥ 140 puntos	57 (38,5%)	91 (61,5%)	148	

No hubo diferencias en cuanto a la mortalidad intrahospitalaria. En el grupo con intervalo QTc normal hubo 15 defunciones (3,8%), mientras que en el grupo de intervalo QTc prolongado hubo 16 defunciones (6,5%) (p=0,123). Asimismo, se evaluó la presencia de arritmias potencialmente mortales: taquicardia ventricular sostenida (TV) y fibrilación ventricular (FV). En el grupo de intervalo QTc normal, 10 pacientes (2,6%) presentaron TV o FV, y en el grupo de intervalo QTc prolongado 9 pacientes (3,7%) presentaron estas arritmias, sin encontrar diferencias significativas al respecto (p=0,419).

### Enfermedad arterial coronaria e intervalo QTc

Del total de pacientes incluidos en el estudio se realizó coronariografía en 602 de ellos (94,9%), 230 pertenecieron al grupo de QTc ≥ 440 ms y 372 al grupo de QTc < 440 ms, de los cuales 571 pacientes (94,8%) tuvieron enfermedad arterial coronaria. De acuerdo con el número de vasos coronarios con lesiones significativas los pacientes fueron clasificados en aquellos con enfermedad de 1, 2 o 3 vasos, tras realizar la comparación entre los dos grupos de estudio según QTc, no se encontraron diferencias significativas (p=0,064), pero con respecto a la arteria responsable del infarto, sí se encontraron diferencias significativas únicamente para la arteria descendente anterior (p=0,041) (Tabla 4).

**Tabla 4:** Enfermedad coronaria e intervalo QTc

Número de vasos enfermos	QTc < 440 ms (n=372)	QTc ≥ 440 ms (n=230)	Total	Valor p
0	22 (5,9%)	9 (3,9%)	31	0,064
1	111 (29,8%)	50 (21,7%)	161	
2	95 (25,5%)	74 (32,3%)	169	
3	144 (38,7%)	97 (42,2%)	241	
Arteria culpable del infarto				
Descendente anterior	281 (75,5%)	190 (82,6%)	471	0,041
Circunfleja	221 (59,4%)	141 (61,3%)	362	0,644
Coronaria derecha	231 (62,1%)	158 (68,7%)	389	0,100
Tronco coronario	51 (13,7%)	23 (10%)	64	0,178

## DISCUSION

En la presente investigación encontramos que los pacientes con IAMSEST e intervalo QTc menor de 440 ms presentan menor puntaje en la escala GRACE lo que significa que la proporción de pacientes de bajo riesgo fue mayor en este grupo en comparación a aquellos que tenían el intervalo QTc ≥ 440 ms (74,1% vs 25,9%, valor p=0,001). Este hallazgo es esperable, ya que se sabe que a mayor isquemia miocárdica habrá más alteraciones eléctricas las cuales se reflejaran en el electrocardiograma de 12 derivadas.

Un intervalo QTc corregido <440 ms no solo se asoció a mayor proporción de pacientes de bajo riesgo según la escala GRACE, sino también a mayor nivel en el valor de troponina I y de NT - pro BNP, así como menor FEVI. Previamente, ya se había reportado que un intervalo QTc prolongado se asocia con menor FEVI ^9^^,^^11^ y a un mayor incremento en el valor de troponinas de alta sensibilidad ^8^^,^^9^. Tanto la FEVI reducida como el mayor valor de troponinas son factores de riesgo reconocidos que incrementan la mortalidad, por ello se puede afirmar que los pacientes con intervalo QTc prolongado tienen mayor riesgo cardiovascular, algo que era de esperarse tras la asociación encontrada entre el mencionado intervalo y la escala de riesgo GRACE.

Pese a que los hallazgos anteriormente descritos parecen redundar con lo ya reportado por otros autores, es importante señalar que uno de los hallazgos novedosos de la presente investigación es que en el grupo con intervalo QTc ≥ 440 ms hubo mayor proporción de pacientes en donde la arteria culpable era la descendente anterior (82,6 75,5%, valor p=0,04). 

La escala de riesgo GRACE predice la mortalidad intrahospitalaria a los 6 meses luego de un infarto agudo de miocardio, y está recomendada para la estratificación de riesgo en el IAMSEST ^2^. Sin embargo, en algunos casos, aún no se conoce el tiempo óptimo de tratamiento intervencionista en estos pacientes, los resultados en los estudios que comparan el tratamiento temprano vs. tardío son controversiales ^1^. Si bien el intervalo QTc no está incluido en la escala GRACE, en este estudio se encontró relación significativa en los pacientes de bajo riesgo.

La medición del intervalo QTc ofrece mucha información pronóstica en diversos escenarios, en la población general un intervalo QTc prolongado puede estar asociado al desarrollo de enfermedad coronaria ^4^^,^^7^; para pacientes con enfermedad coronaria ya establecida la prolongación del QT incrementa el riesgo de muerte súbita ^5^^,^^6^, mientras que en el contexto de un IAM el incremento del mencionado intervalo se asocia con mayor mortalidad tanto en pacientes con o sin elevación del segmento ST ^9^^,^^10^. En el escenario de un infarto agudo de miocardio (IAM), se desconoce cuál es la mejor fórmula para corregir el intervalo QT, si bien la fórmula de Bazett es la más popular y la más ampliamente usada, esta parece sobrestimar el intervalo QTc ^13^; existen discrepancia con respecto a qué fórmula usar en el escenario de un IAMSEST. Dos estudios, uno realizado en España y otro en Irán, utilizaron la fórmula de Bazett ^8^^,^^11^, mientras que otro, realizado en India, utilizó la fórmula de Hogdes ^9^.

Dos estudios en un seguimiento a 30 días de pacientes con IAMSEST, encontraron asociación entre el intervalo QTc y eventos cardiovasculares mayores: a mayor intervalo QTc mayor mortalidad cardiovascular ^8^^,^^9^. En uno de estos estudios se encontró que un intervalo QTc > 468 ms predijo eventos cardiovasculares mayores con una sensibilidad de 72% y una especificidad de 61% ^9^, mientras que, en el otro, el punto de corte fue > 458 ms con una sensibilidad de 76,2% y una especificidad de 88,2% ^8^.

Con base en lo anterior, parece ser que el intervalo QTc corregido tiene poder para predecir la mortalidad en un seguimiento de, al menos, 30 días; sin embargo, en otro estudio publicado por Nabati *et al.* que incluyó a 205 pacientes con IAMSEST y con angina inestable, no se encontró diferencias significativas en cuanto a la mortalidad intrahospitalaria, pero no se hizo un seguimiento a los 30 días ^11^. En nuestro caso, a pesar de haber incluido un mayor número de pacientes, tampoco encontramos diferencias en cuanto a la mortalidad intrahospitalaria.

Tradicionalmente la isquemia se ha asociado únicamente con alteraciones en el segmento ST (elevación o depresión > 1 mm); sin embargo, estas alteraciones no son las únicas ^14^ ni tampoco necesariamente las primeras ^15^. Un estudio demostró que la prolongación del intervalo QTc precede a las alteraciones del ST en el escenario de isquemia miocárdica aguda ^15^. El mecanismo por el cual la isquemia prolonga el intervalo QT es por los cambios bifásicos en la duración del potencial de acción (prolongación inicial seguido de acortamiento), cambios que ocurren de manera precoz; además, la isquemia retrasa el proceso de despolarización y repolarización de forma más significativa ^15^. Por lo tanto, a mayor isquemia miocárdica, mayor prolongación del intervalo QTc y, consecuentemente, peor pronóstico.

Estudios previos han demostrado la correlación entre el intervalo QTc y la escala de riesgo TIMI (*Thrombolysis in Myocardial Infartion*) ^8^^,^^9^^,^^11^, pero no se ha estudiado la relación con la escala de riesgo GRACE, la cual es de uso más extendido en el IAMSEST. Dada la heterogeneidad de presentación de esta enfermedad, el desarrollo de escalas de riesgo novedosas con la inclusión de este intervalo podría proporcionar al clínico información valiosa para la toma oportuna de decisiones; sin embargo, se necesitan más estudios controlados que validen nuestros resultados.

La presente investigación tiene varias limitaciones, primero se trata de un estudio retrospectivo unicéntrico, motivo por el cual los resultados encontrados no se podrían extrapolar a otras poblaciones. Segundo existen diferencias en el intervalo QTc entre ambos sexos, las mujeres suelen tener valores ligeramente mayores a la de los varones, sin embargo, en el presente estudio se consideró el mismo punto de corte del intervalo QTc para ambos sexos. Tercero la evaluación de la mortalidad está infraestimada ya que solo evaluamos la mortalidad durante la estancia hospitalaria pero no realizamos un seguimiento a largo plazo de los pacientes. Finalmente, solo se consideraron pacientes admitidos en la Unidad Coronaria, es probable que algunos pacientes hayan tenido IAMSEST de bajo riesgo y hayan recibido manejo ambulatorio.

En conclusión, un intervalo QTc normal (<440 ms) se relaciona con una escala de riesgo GRACE de riesgo bajo en pacientes con IAMSEST. Además, se encontró una relación entre el intervalo QT prolongado y el compromiso de la arteria descendente anterior
